# Data on introduced plants in Zimbabwe: Floristic changes and patterns of collection based on historical herbarium records

**DOI:** 10.1016/j.dib.2017.09.046

**Published:** 2017-09-22

**Authors:** Alfred Maroyi

**Affiliations:** Medicinal Plants and Economic Development (MPED) Research Center, Department of Botany, University of Fort Hare, Private Bag X1314 Alice 5700, South Africa

**Keywords:** Casual, Floristic changes, Invasive, Naturalised, National herbarium, Zimbabwe

## Abstract

National herbaria with significant historical plant collections are critical to tracking floristic changes and patterns, which include the introduction and spread of non-native plant species. To explore the importance of herbarium specimen data in understanding floristic changes in Zimbabwe, the plant collections housed by the National Herbarium (SRGH) in Harare, Zimbabwe were utilized with historical specimens dating back to 1870. A list of naturalised plant taxa and collection data were compiled. A total of 2916 plant specimens were recorded, comprising of 401 taxa, 237 genera and 76 plant families. Twenty eight specimens (1.0%) were collected between 1870 and 1908, prior to the establishment of the National Herbarium in 1909 and 123 specimens (4.2%) were collected in the first 25 years of the establishment of the institute (1909–1934). Intensive collection of herbarium specimens of casual, naturalised and invasive alien plant species occurred between 1950 and 1970. This data demonstrates the utility of plant species data housed in the National Herbaria and how such data can be used to map floristic changes and patterns.

**Specifications Table**

**Table:** 

Subject area	*Biology*
More specific subject area	*Botany*
Type of data	*Tables, text file and graph*
How data was acquired	*Surveys, conducting herbarium studies by documenting exotic plant species housed by the National Herbarium, Harare, Zimbabwe*
Data format	*Raw, filtered and analyzed*
Experimental factors	*All parameters of available data were taken and processed based on 2916 herbarium records*
Experimental features	*Herbarium specimen retrieval*
Data source location	*Harare, Zimbabwe*
Data accessibility	*The data are available with this article*

**Value of the data**

• The data are critical in tracking floristic changes and patterns of spread of exotic plant species.
• This data are important for monitoring purposes and also to fill the gaps in plant distributional ranges.
• This data will contribute to better understanding of the ecological impacts of exotic plant species.
• List of herbarium specimens of exotic plant species will help in future research on this category of plant species.

## 1 Data

Many non-native plants in Zimbabwe were introduced decades ago through agriculture and horticulture. A number these exotics now occupy large stretches of land and form characteristic features of the Zimbabwean flora. The number of plant exotics increase with time, 1449 exotic taxa have been recorded in Zimbabwe, a country comprising about 6000 plant species [1]. But only a handful of exotic plant species are represented in local herbaria, although most of these species are known to be widely distributed than indicated in herbaria. This development is unfortunate as herbaria must provide quality specimens and data that represent an overview of floristic changes over time. Herbarium specimens can also be used to document plant distributional changes such as exotic species expansions. Periodic plant collecting expeditions are important for monitoring purposes and also to fill the gaps in plant distributional ranges. Collection of herbarium specimens helps in the identification of invasive exotics and detection of new introductions.

The herbarium specimens usually contain important historical information about plant species which is critical for scientific studies. Such information on herbarium labels includes the identity of the species and other details such as family, habit, plant height, leaf arrangement, shape, size, flower colour, shape and size. Other important information usually included on the herbarium label include details about the collector, his or her name, collector’s number, the locality, year of collection of the plant species, description of the habitat and notes on uses of the plant species if any and also size of the population. For exotic species, experienced collectors usually include information such as life history traits, such as habit, population size and perceived invasion status, whether casual, naturalised or invasive. Pyšek et al. [2] defined a casual alien as a plant species that reproduces occasionally outside cultivation and it does not form self-sustaining populations and relying on repeated introductions for its persistence. According to Pyšek et al. [2], a naturalised species is defined as an alien species that reproduces consistently without direct human intervention, and invasive alien species as a naturalised species that produces offspring in large numbers and at considerable distances from the parent plants with the potential to spread over a large area. Studies of the distributional history of non-native plant species can provide us with the knowledge of their earliest locations, patterns of colonization and rates of spread. A better understanding of their establishment and distributional changes over time is vital for making informed decisions in managing existing introductions and in predicting future invasions. To explore the importance of herbarium specimen data for understanding floristic changes and patterns in Zimbabwe with regards to non-native plants, herbarium specimens collected in the country that are housed in the National Herbarium of Zimbabwe, Harare were inventoried. The first herbarium records and subsequent temporal collection of each taxa were identified and recorded.

## 2 Experimental design, materials and methods

Included in this data are those plant species considered to be naturalized in Zimbabwe [1], [3], [4]. Therefore, a database of casual, naturalised and invasive alien flora [2], occurring in Zimbabwe was compiled based on herbarium records. All alien plant species ever recorded in Zimbabwe as escapes from cultivation or naturalised at least once in the wild, and housed by the National Herbarium, Harare, Zimbabwe (SRGH, acronym adhere to Index Herbariorum) [5], were included in the database. Plant species introduced in Zimbabwe or cultivated without any evidence of having escaped were not considered. Information on herbarium labels, including locality and year of collection of the plant species, was examined and recorded. For each taxon, information on year when the plant species was first collected, mode or purpose of introduction, its invasion status in Zimbabwe and subsequent temporal collection data were extracted from the herbarium records. The mode or purpose of introduction was included, and also whether the introduction was accidental or intentional. The correct identities and names of all the taxa listed were checked and, where names have changed, the currently accepted name was applied. Therefore, plant taxa name, families and plant authorities were verified using taxonomical floras, journal articles and internet sources such as the International Plant Name Index (www.ipni.org), the Missouri Botanical Garden's Tropicos Nomenclatural database (www.tropicos.org) and the Royal Botanic Garden and Missouri Botanic Garden plant name database (www.theplantlist.org).

**Fig. 1:**
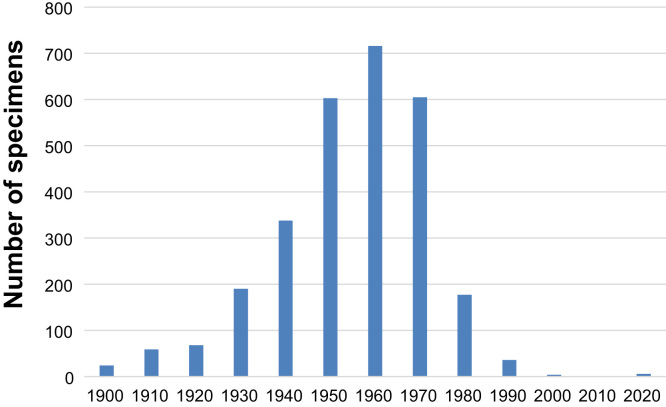
The number of herbarium specimens of exotic plant species collected in each decade of the National Herbarium’s history.

A total of 2916 plant specimens were recorded, comprising of 401 taxa, 237 genera and 76 plant families (Table 1). Appendix A provides an alphabetical listing of plants taxa. Close to three quarters of these taxa (77.6%) belong to 19 families given in Table 2. Pant families Asteraceae, Poaceae and Solanaceae accounted for the highest number of species (Table 2). A significant proportion of the exotics (61.6%) are represented in the National Herbarium by between one to five specimens (Table 3). Twenty eight specimens (1.0%) were collected between 1870 and 1908, prior to the establishment of the National Herbarium in 1909 and 123 specimens (4.2%) were collected in the first 25 years of the establishment of the institute (1909–1934) (Fig. 1). Most of the herbarium specimens were collected between 1930 and 1980 (Fig. 1). Intensive collection of herbarium specimens of casual, naturalised and invasive alien plant species occurred between 1950 and 1970 with reduced collecting activity in the 1990s and 2010 (Fig. 1). A total of 1184 herbarium specimens representing 231 taxa (57.6%) of the exotic species pre-date 1950. By 1970, 2500 herbarium specimens representing 358 taxa (89.3%) had been collected (Appendix A). Results of the present study are important in understanding distributional changes of introduced plant species in Zimbabwe.

**Table 1:** Total number of casual, naturalised and invasive taxa, genera and plant families in Zimbabwe.

**Plant group**	**No. of families**	**No. of genera**	**No. of taxa**
Pteridophytes	5	5	5
Gymnosperms	2	2	6
Monocotyledons	8	39	62
Dicotyledons	61	191	328
**Total**	**76**	**237**	**401**

**Table 2:** Families with five or more casual, naturalised and invasive taxa in Zimbabwe.

**Family**	**No. of taxa**	**%**
Asteraceae	53	13.2
Fabaceae	51	12.7
Poaceae	49	12.2
Solanaceae	21	5.2
Brassicaceae	15	3.7
Amaranthaceae	14	3.5
Convolvulaceae	13	3.2
Euphorbiaceae	13	3.2
Myrtaceae	11	2.7
Boraginaceae	10	2.5
Polygonaceae	10	2.5
Chenopodiaceae	8	2.0
Malvaceae	8	2.0
Verbenaceae	8	2.0
Caryophyllaceae	7	1.7
Lamiaceae	7	1.7
Onagraceae	5	1.2
Passifloraceae	5	1.2
Plantaginaceae	5	1.2

**Table 3:** Proportion of plant exotics represented by between one to five specimens in the National Herbarium.

**No. of collections**	**No. of taxa**	**%**
One specimen	84	20.9
Two specimens	70	17.5
Three specimens	40	10.0
Four specimens	25	6.2
Five specimens	28	7.0
**Total**	**247**	**61.6**

## Figures and Tables

**Table:** 

**Species**	**Family**	**Reasons for introduction**	**Current status**	**Earliest herbarium record**	**<1934**	**1935-1960**	**1961-1986**	**1987>**	**Total herbarium specimens**
Abelmoschus esculentus (L.) Moench	Malvaceae	Food, Fibre	Casual	1933	1	3			4
Acacia baileyana F.Muell.	Fabaceae	Ornamental	Casual	1941		1	1		2
Acacia dealbata Link	Fabaceae	Ornamental, tanbark	Naturalised	1902	1		4		5
Acacia decurrens Willd.	Fabaceae	Ornamental, tanbark	Naturalised	1903	1		3		4
Acacia elata Benth.	Fabaceae	Ornamental	Naturalised	1944		1	1		2
Acacia longifolia (Andrews) Willd.	Fabaceae	Ornamental, timber	Casual	1932	1		2		3
Acacia mearnsii De Wild.	Fabaceae	Ornamental, tanbark, timber	Invasive	1931	2	3	8		13
Acacia melanoxylon R.Br.	Fabaceae	Ornamental, timber	Invasive	1903	1		6		7
Acacia podalyriifolia G.Don	Fabaceae	Ornamental	Naturalised	1971			1		1
Acacia salicina Lindl.	Fabaceae	Ornamental	Casual	1993				1	1
Acalypha amentacea subsp. wilkesiana (Müll.Arg.) Fosberg.	Euphorbiaceae	Ornamental	Casual	1974			4		4
Acanthospermum australe (Loefl.) Kuntze	Asteraceae	Unintentional	Invasive	1931	3	11	8	1	23
Acanthospermum glabratum (DC.) Wild	Asteraceae	Unintentional	Naturalised	1951		2	5		7
Acanthospermum hispidum DC.	Asteraceae	Unintentional	Invasive	1917	1	15	11		27
Achillea millefolium L.	Asteraceae	Medicinal	Casual	1942		1	2		3
Achyranthes aspera L. var. pubescens (Moq.) C.C. Towns	Amaranthaceae	Unintentional	Invasive	1906	10	13	16	3	42
Achyranthes aspera L. var. sicula L.	Amaranthaceae	Unintentional	Invasive	1941		4	2		6
Adiantum raddianum C. Presl	Pteridaceae	Ornamental	Naturalised	1905	2	10	4		18
Agave americana L.	Asparagaceae	Fibre, ornamental	Naturalised	NA					
Agave sisalana Perrine	Asparagaceae	Fibre, ornamental	Naturalised	NA					
Ageratina adenophora (Spreng.) King & H. Rob.	Asteraceae	Ornamental	Invasive	1945		1	1	1	3
Ageratum conyzoides L.	Asteraceae	Ornamental	Invasive	1943		1	11	2	14
Ageratum houstonianum Mill.	Asteraceae	Ornamental	Naturalised	1955		1			1
Albizia procera (Roxb.) Benth.	Fabaceae	Ornamental	Casual	1929	1		3		4
Alisma plantago-aquatica L.	Alismataceae	Ornamental	Naturalised	1972			1		1
Alternanthera caracasana Kunth	Amaranthaceae	Unintentional	Naturalised	1931	2	2	3		7
Alternanthera nodiflora R.Br.	Amaranthaceae	Unintentional	Naturalised	1921	1	20	26		47
Alternanthera pungens Kunth	Amaranthaceae	Unintentional	Naturalised	1931	2	6	17	1	26
Alternanthera sessilis (L.) R. Br. ex DC.	Amaranthaceae	Unintentional	Naturalised	1946		4	20	2	26
Amaranthus dubius Mart. ex Thell.	Amaranthaceae	Unintentional	Casual	1960		1	1		2
Amaranthus hybridus L. ssp. cruentus (L.) Thell.	Amaranthaceae	Food	Naturalised	1952		2	1	1	4
Amaranthus hybridus subsp. hybridus	Amaranthaceae	Food	Naturalised	1918	6	8	13	1	28
Amaranthus spinosus L.	Amaranthaceae	Food	Invasive	1931	1	9	17	1	28
Amaranthus viridis L.	Amaranthaceae	Food	Invasive	1942		1	4		5
Ambrosia maritima L.	Asteraceae	Unintentional	Naturalised	1946		3			3
Amsinckia menziesii (Lehm.) A. Nelson & J.F. Macbr.	Boraginaceae	Unintentional	Casual	1980			1		1
Anagallis arvensis L. ssp. arvensis	Primulaceae	Unintentional	Casual	1953		1	1		2
Apium leptophyllum (DC.) F.Muell.	Apiaceae	Unintentional	Naturalised	1923	1	12	4		17
Arachis hypogea L., 1753	Fabaceae	Food	Casual	1952		1			1
Argemone mexicana L.	Papaveraceae	Unintentional	Invasive	1918	4	8	18		30
Aristolochia littoralis D. Parodi	Aristolochiaceae	Ornamental	Casual	1968			1		1
Arundo donax L.	Poaceae	Ornamental	Invasive	1958		1			1
Asclepias curassavica L.	Apocynaceae	Ornamental	Casual	1965			2		2
Avena fatua L.	Poaceae	Fodder	Casual	1958		2	5		7
Avena sativa L.	Poaceae	Food	Casual	1975			4		4
Avena sterilis subsp. ludoviciana	Poaceae	Fodder	Casual	1969			5		5
Axonopus compressus (Sw.) P.Beauv.	Poaceae	Ornamental	Naturalised	1976			1		1
Axonopus fissifolius (Raddi) Kuhlm.	Poaceae	Ornamental	Naturalised	1943		2	2	1	5
Azolla filiculoides Lam.	Azollaceae	Ornamental	Invasive	1983			1	1	2
Bambusa vulgaris Schrad.	Poaceae	Ornamental	Casual	1961			1		1
Bauhinia variegata L. var. candida Voigt	Fabaceae	Ornamental	Naturalised	1923	4	8	1		13
Bidens bipinnata L.	Asteraceae	Unintentional	Invasive	1969			1		1
Bidens biternata (Lour.) Merr. & Sherff	Asteraceae	Unintentional	Naturalised	1920	1	15	6		22
Bidens pilosa L.	Asteraceae	Unintentional	Invasive	1906	6	13	12	1	32
Boerhavia coccinea Mill.	Nyctagineceae	Unintentional	Casual	1933	1	9	12		32
Boerhavia diffusa L.	Nyctagineceae	Unintentional	Naturalised	1906	5	8	11		24
Boerhavia erecta L.	Nyctagineceae	Unintentional	Naturalised	1977			1	1	2
Brachiaria mutica (Forssk.) Stapf	Poaceae	Ornamental	Casual	1957		1	1		2
Brachiaria ramosa (L.) Stapf	Poaceae	Fodder	Casual	1976			2		2
Brachiaria umbellata (Trin.) Clayton	Poaceae	Ornamental	Casual	1962			1		1
Brassica juncea (L.) Czern.	Brassicaceae	Food	Casual	1912	1	8	2		11
Brassica rapa L.	Brassicaceae	Food	Casual	1941		2			2
Briza maxima L.	Poaceae	Unintentional	Naturalised	1925	1	1	5		7
Briza minor L.	Poaceae	Unintentional	Naturalised	1931	2	1			3
Bromus catharticus Vahl	Poaceae	Fodder	Naturalised	1922	6	7	11	1	25
Bromus diandrus Roth	Poaceae	Ornamental	Casual	1977			1		1
Bromus hordeaceus L. ssp. ferronii (Mabille) P.M. Sm.	Poaceae	Fodder	Casual	1969			1		1
Bromus leptocladus Nees	Poaceae	Fodder	Casual	1931	1	6	3	1	11
Browallia americana L.	Solanaceae	Ornamental	Invasive	1954		1	2		3
Bryophyllum proliferum Bowie ex Hook.	Crassulaceae	Ornamental	Casual	1931	1	2	1		4
Bryophyllum tubiflorum Harv.	Crassulaceae	Ornamental	Naturalised	1947		1			1
Buddleja davidii Franch.	Scrophulariaceae	Ornamental	Casual	1949		1			1
Caesalpinia decapetala (Roth) Alston	Fabaceae	Ornamental	Naturalised	1925	2	5	5		12
Cajanus cajan (L.) Millsp.	Fabaceae	Food	Casual	1959		1	2		3
Calceolaria tripartita Ruiz & Pav.	Calceolariaceae	Ornamental	Casual	1949		1	1		2
Callistemon viminalis (Sol. ex Gaertn.) G.Don	Myrtaceae	Ornamental	Naturalised	1956		1			1
Callitris calcarata F.Muell.	Cupressaceae	Ornamental, Timber	Naturalised	1908	1	2			3
Calotropis procera (Aiton) W.T. Aiton	Apocynaceae	Ornamental	Naturalised	1951		1	1	1	3
Canna indica L.	Cannaceae	Ornamental	Naturalised	1929	1	3	1		5
Cannabis sativa L.	Cannabaceae	Unintentional	Casual	1912	1	4	4		9
Capsella bursa-pastoris (L.) Medik.	Brassicaceae	Food	Naturalised	1942		5	2		7
Capsicum annuum var. glabriusculum (Dunal) Heiser & Pickersgill	Solanaceae	Food	Casual	1948		3	2		5
Cardamine flexuosa With.	Brassicaceae	Food	Naturalised	1944		2	3		5
Cardamine hirsuta L.	Brassicaceae	Unintentional	Casual	1952		1			1
Cardiospermum grandiflorum Sw.	Sapindiaceae	Ornamental	Naturalised	1947		1			1
Catharanthus roseus (L.) G. Don	Apocynaceae	Ornamental	Casual	1932	1	6	3		10
Centaurea melitensis L.	Asteraceae	Ornamental	Naturalised	1953		1	1		2
Centratherum punctatum Cass. ssp. punctatum	Asteraceae	Ornamental	Naturalised	1958		1	1		2
Centrosema pubescens Benth.	Fabaceae	Unintentional	Casual	1912	1	6	6		13
Cerastium glomeratum Thuill.	Caryophyllaceae	Unintentional	Casual	1955		3			3
Cestrum aurantiacum Lindl.	Solanaceae	Ornamental	Naturalised	1959		1			1
Chamaecrista rotundifolia (Pers.) Greene	Fabaceae	Unintentional	Naturalised	1955		1	1		2
Chenopodium album L.	Chenopodiaceae	Food	Invasive	1922	3	6	2	1	12
Chenopodium giganteum D. Don	Chenopodiaceae	Unintentional	Casual	1936		3	2		5
Chenopodium hederiforme (Murray) Aellen	Chenopodiaceae	Unintentional	Casual	1956		2		1	3
Chenopodium murale L.	Chenopodiaceae	Unintentional	Naturalised	1931	2	8	3		13
Chenopodium opulifolium Schrad. ex W. D. J. Koch & Ziz	Chenopodiaceae	Unintentional	Naturalised	1931	2	1	3		6
Chromolaena odorata (L.) R.M. King & H. Rob.	Asteraceae	Ornamental	Casual	1967			1		1
Cichorium intybus L.	Asteraceae	Food, medicinal, ornamental	Casual	1944		2	2		4
Citrus aurantium L.	Rutaceae	Food	Casual	1947		1	1		2
Citrus limon (L.) Burm. f.	Rutaceae	Food	Casual	1916	2	3	2		7
Cobaea scandens Cav.	Cobaeaceae	Unintentional	Naturalised	1957		1	2		3
Coffea arabica L.	Rubiaceae	Food	Naturalised	1927	2	2	3		7
Colocasia esculenta (L.) Schott	Araceae	Food	Naturalised	1962			8		8
Conium maculatum L.	Apiaceae	Food	Casual	1974			1		1
Coreopsis lanceolata L.	Asteraceae	Ornamental	Invasive	1975			3		3
Corymbia citriodora (Hook.) K.D.Hill & L.A.S.Johnson	Myrtaceae	Timber	Naturalised	1904	1	1			2
Cosmos bipinnatus Cav.	Asteraceae	Ornamental	Naturalised	1931	1	2	1		4
Cosmos sulphureus Cav.	Asteraceae	Ornamental	Casual	1932	1	4	5		10
Cotoneaster pannosus Franch.	Rosaceae	Ornamental	Naturalised	1965			1		1
Crotalaria distans Benth. ssp. macrotropis (Baker f.) Polhill	Fabaceae	Green Manure	Casual	1920	1	1			2
Crotalaria juncea L.	Fabaceae	Fibre	Naturalised	1945		1	1		2
Crotalaria pallida Aiton var. pallida	Fabaceae	Green Manure	Naturalised	1936		7	1		8
Cucumis anguria L. var. anguria	Cucurbitaceae	Food	Naturalised	1932	1	1	1		3
Cucumis melo L.	Curcurbitaceae	Food	Casual	1953		1			1
Cupressus lusitanica Mill.	Cupressaceae	Ornamental, Timber	Invasive	1900	1	2	1		4
Cupressus torulosa D.Don	Cupressaceae	Ornamental, Timber	Casual	1895	1	1			2
Cuscuta campestris Yunck.	Convolvulaceae	Unintentional	Invasive	1948		7	10	1	18
Cuscuta suaveolens Ser.	Convolvulaceae	Unintentional	Naturalised	1936		1	1		2
Cylindropuntia fulgida (Engelm.) F.M.Knuth	Cactaceae	Ornamental	Invasive	1973			1		1
Cynodon aethiopicus Clayton & Harlan	Poaceae	Fodder	Casual	1931	1	2	13		16
Cynodon nlemfuensis Vanderyst var. nlemfuensis	Poaceae	Fodder	Casual	1917	6	4	17	1	28
Cynodon nlemfuensis Vanderyst var. robustus Clayton & Harlan	Poaceae	Fodder	Casual	1922	3	4			4
Cyperus rotundus L.	Cyperaceae	Unintentional	Casual	1971			1		1
Dactylis glomerata L.	Poaceae	Fodder	Casual	1965			4		4
Dahlia pinnata Cav.	Asteraceae	Ornamental	Casual	1970			1		1
Dalbergia sissoo DC.	Fabaceae	Ornamental	Naturalised	1910	5	4	8		17
Datura inoxia P. Mill.	Solanaceae	Ornamental	Invasive	1957		3	3	1	7
Datura quercifolia Kunth	Solanaceae	Ornamental	Invasive	1941		9			9
Datura stramonium L.	Solanaceae	Ornamental	Invasive	1906	2	7	1		10
Desmodium tortuosum (Sw.) DC.	Fabaceae	Green Manure	Naturalised	1919	3	4	7		14
Desmodium triflorum (L.) DC.	Fabaceae	Unintentional	Naturalised	1935	1	1			2
Desmodium uncinatum (Jacq.) DC.	Fabaceae	Unintentional	Naturalised	1967			5		5
Dichanthium aristatum (Poir.) C.E.Hubb.	Poaceae	Fodder	Casual	1920	2				2
Dichondra micrantha Urb.	Convolvulaceae	Unintentional	Invasive	1945		1	2		3
Digitalis purpurea L.	Plantaginaceae	Ornamental	Casual	1969			1		1
Dolichandra unguis-cati (L.) L.G. Lohmann	Bignoniaceae	Ornamental	Naturalised	1958		1	1		2
Dolichandra unguis-cati (L.) L.G. Lohmann	Bignoniaceae	Ornamental	Casual	1967			1		1
Duranta erecta L.	Verbenaceae	Ornamental	Invasive	1905	5	16	18		39
Dysphania ambrosioides (L.) Mosyakin & Clemants	Chenopodiaceae	Unintentional	Invasive	1906	5	16	20	1	42
Dysphania carinata (R. Br.) Mosyakin & Clemants	Chenopodiaceae	Unintentional	Naturalised	1939		1	3		4
Dysphania schraderiana (Schult.) Mosyakin & Clemants	Chenopodiaceae	Unintentional	Naturalised	1920	3	7	2		12
Echinochloa crus-galli var. crus-galli (L.) P. Beauv.	Poaceae	Unintentional	Casual	1970			2		2
Echinochloa frumentacea Link	Poaceae	Ornamental	Casual	1944		5	3		8
Echium plantagineum L.	Boraginaceae	Ornamental	Naturalised	1918	3		2		5
Eclipta prostrata (L.) L.	Asteraceae	Unintentional	Invasive	1931	1		1		2
Egeria densa Planch.	Hydrocharitaceae	Ornamental	Casual	1950		1	1		2
Eichhornia crassipes (Mart.) Solms	Pontederiaceae	Ornamental	Invasive	1944		4	5		9
Emex australis Steinh.	Polygonaceae	Unintentional	Casual	1922	1	2			3
Eragrostis tef (Zucc.) Trotter	Poaceae	Food	Casual	1912	7	2	4		13
Erigeron canadensis L.	Asteraceae	Unintentional	Invasive	1903	1	4	6		11
Erigeron floribundus (Kunth) Sch. Bip.	Asteraceae	Unintentional	Invasive	1931	3	8	12		23
Erigeron karvinskianus DC.	Asteraceae	Ornamental	Invasive	1967			4		4
Erigeron primulifolius (Lam.) Greuter	Asteraceae	Unintentional	Invasive	1932	1	7	11		19
Eruca vesicaria subsp. sativa (Mill.) Thell.	Brassicaceae	Food	Casual	1956		1	2		3
Erucastrum arabicum Fisch. & C.A. Mey. var. arabicum	Brassicaceae	Unintentional	Casual	1912	1		1		2
Eucalyptus camaldulensis Dehnh.	Myrtaceae	Timber	Naturalised	1897	1	2	3	1	7
Eucalyptus globulus Labill.	Myrtaceae	Timber	Invasive	1971			1		1
Eucalyptus grandis W. Hill ex Maiden	Myrtaceae	Timber	Naturalised	1957		3			3
Eucalyptus robusta Sm.	Myrtaceae	Timber	Naturalised	1969			1		1
Eucalyptus tereticornis Sm.	Myrtaceae	Timber	Naturalised	1908	1	1			2
Euphorbia cyathophora Murray	Euphorbiaceae	Ornamental	Invasive	1924	1	1			2
Euphorbia heterophylla L.	Euphorbiaceae	Unintentional	Invasive	1956		1	1		2
Euphorbia hirta L.	Euphorbiaceae	Unintentional	Invasive	1917	5	10	11		26
Euphorbia indica Lam.	Euphorbiaceae	Unintentional	Invasive	1948		1	1		2
Euphorbia peplus L.	Euphorbiaceae	Unintentional	Naturalised	1953		1	1		2
Euphorbia prostrata Aiton	Euphorbiaceae	Unintentional	Invasive	1931	2	8	7		17
Euphorbia serpens Kunth	Euphorbiaceae	Unintentional	Invasive	1922	3	3	3	3	11
Euphorbia tirucalli L.	Euphorbiaceae	Ornamental	Invasive	1931	1	2	1		4
Euryops chrysanthemoides (DC.) B. Nord.	Asteraceae	Ornamental	Naturalised	1942		1	1		2
Fagopyrum esculentum Moench.	Polygonaceae	Unintentional	Casual	1919	2	3	3		8
Flaveria bidentis (L.) Kuntze	Asteraceae	Unintentional	Naturalised	1949		1	2		3
Flaveria trinervia (Spreng.) C. Mohr	Asteraceae	Unintentional	Invasive	1931	3	8	20		31
Foeniculum vulgare Mill.	Apiaceae	Food	Casual	1958		1			1
Fumaria muralis W.D.J.Koch	Papaveraceae	Ornamental	Naturalised	1956		1			1
Galinsoga parviflora Cav.	Asteraceae	Unintentional	Invasive	1926	4	12	11	2	29
Galinsoga quadriradiata Ruiz & Pav.	Asteraceae	Unintentional	Naturalised	1934	1	1			2
Gnaphalium purpureum L.	Asteraceae	Unintentional	Invasive	1950		7	6		13
Gomphocarpus physocarpus E. Mey.	Apocynaceae	Ornamental	Naturalised	1942		1	1		2
Gomphrena celosioides Mart.	Amaranthaceae	Unintentional	Invasive	1917	2	10	12		24
Gomphrena globosa L.	Amaranthaceae	Unintentional	Casual	1955		1	1		2
Gossypium hirsutum L.	Malvaceae	Fibre	Casual	1927	1	1			2
Grevillea robusta A. Cunn. ex R. Br.	Proteaceae	Ornamental, Timber	Invasive	1976			1		1
Guilleminea densa (Humb. & Bonpl. ex Schult.) Moq.	Amaranthaceae	Unintentional	Invasive	1959		1	9		10
Harpachne schimperi A. Rich.	Poaceae	Unintentional	Naturalised	1991				1	1
Hedychium gardnerianum Sheppard ex Ker Gawl.	Zingiberaceae	Unintentional	Naturalised	1968			1		1
Heliotropium amplexicaule Vahl	Boraginaceae	Unintentional	Casual	1942		1	1		2
Heliotropium indicum L.	Boraginaceae	Unintentional	Naturalised	1953		3	6		9
Holcus lanatus L.	Poaceae	Unintentional	Casual	1950		1			1
Homalanthus populifolius Graham	Euphorbiaceae	Ornamental	Naturalised	1943		3	3		6
Hydrocotyle ranunculoides L.f.	Apiaceae	Unintentional	Casual	1964			1		1
Hypochaeris radicata L.	Asteraceae	Unintentional	Naturalised	1952		1	4		5
Hyptis pectinata (L.) Poit.	Lamiaceae	Unintentional	Naturalised	1963			1		1
Hyptis spicigera Lam.	Lamiaceae	Unintentional	Casual	1955		1	1		2
Hyptis suaveolens (L.) Poit.	Lamiaceae	Unintentional	Casual	1945		1	1		2
Imperata cylindrica (L.) Raeusch.	Poaceae	Unintentional	Casual	1973			1		1
Indigofera microcarpa Desv.	Fabaceae	Unintentional	Naturalised	1930	1	7	6		14
Ipomoea alba L.	Convolvulaceae	Unintentional	Naturalised	1947		1	1		2
Ipomoea batatas (L.) Lam.	Convolvulaceae	Food	Casual	1918	1	1	1		3
Ipomoea carnea subsp. fistulosa (Mart. ex Choisy) D.F. Austin	Convolvulaceae	Ornamental	Naturalised	1947		1	1		2
Ipomoea hederifolia L.	Convolvulaceae	Ornamental	Casual	1978			1		1
Ipomoea indica (Burm.) Merr. var. acuminata (Vahl) Fosberg	Convolvulaceae	Ornamental	Naturalised	1959		1			1
Ipomoea nil (L.) Roth	Convolvulaceae	Unintentional	Casual	1907	1	1	1		3
Ipomoea purpurea (L.) Roth	Convolvulaceae	Ornamental	Invasive	1917	1	1			2
Ipomoea tricolor Cav.	Convolvulaceae	Ornamental	Naturalised	1976			1	1	2
Ipomoea turbinata Lag.	Convolvulaceae	Unintentional	Casual	1952		1	1		2
Jacaranda mimosifolia D. Don	Bignoniaceae	Ornamental	Invasive	1922	1		4		5
Jatropha curcas L.	Euphorbiaceae	Ornamental	Naturalised	1933	1	6	6		13
Jatropha gossypiifolia L. var. elegans (Pohl) Müll-Arg.	Euphorbiaceae	Ornamental	Naturalised	1955		1	1		2
Kalanchoe pinnata (Lam.) Pers.	Crassulaceae	Ornamental	Naturalised	1938		1	1		2
Lactuca serriola L.	Asteraceae	Unintentional	Naturalised	1977			2		2
Lantana camara L.	Verbenaceae	Ornamental	Invasive	1937		12	14	4	30
Lantana trifolia L.	Verbenaceae	Ornamental	Casual	1905	2	5	7		14
Laphangium luteoalbum (L.) Tzvelev	Asteraceae	Unintentional	Invasive	1928	1	2			3
Leontodon saxatilis Lam.	Asteraceae	Unintentional	Casual	1951		1			1
Lepidium bonariense L.	Brassicaceae	Food	Casual	1953		3	8		11
Lepidium didymum L.	Brassicaceae	Food	Naturalised	1953		3	5		8
Leucaena leucocephala (Lam.) de Wit	Fabaceae	Fodder	Casual	1949		1			1
Ligustrum lucidum W.T. Aiton	Oleaceae	Ornamental	Naturalised	1933	1	2	4		7
Ligustrum spp.	Oleaceae	Ornamental	Naturalised	1962			1		1
Lilium formosanum Wallace	Liliaceae	Ornamental	Naturalised	1961			5		5
Linum usitatissimum L.	Linaceae	Unintentional	Casual	1949		1			1
Lolium multiflorum × perenne	Poaceae	Fodder	Casual	1948		2	1		3
Lolium multiflorum Lam.	Poaceae	Fodder	Casual	1967			2		2
Lolium perenne L.	Poaceae	Fodder	Casual	1957		2	5		5
Lolium temulentum L.	Poaceae	Fodder	Casual	1921	4	1			5
Lupinus mexicanus Cerv.	Fabaceae	Unintentional	Naturalised	1963			2		2
Macroptilium atropurpureum (DC.) Urb.	Fabaceae	Fodder	Naturalised	1961			6		6
Malva neglecta Wallr.	Malvaceae	Unintentional	Casual	1959		1			1
Malva parviflora L. var. parviflora	Malvaceae	Unintentional	Casual	1921	1		4		5
Malva sylvestris L.	Malvaceae	Unintentional	Casual	1955		1	3		4
Malva verticillata L.	Malvaceae	Unintentional	Casual	1940		1	4		5
Malvastrum coromandelianum (L.) Garcke	Malvaceae	Unintentional	Invasive	1945		3	4		7
Mangifera indica L.	Anacardiaceae	Food	Casual	1959		2			2
Medicago orbicularis (L.) Bartal.	Fabaceae	Fodder	Casual	1962			1		1
Medicago polymorpha L. var. brevispica (Benth.) Heyn	Fabaceae	Fodder	Casual	1910	7	2	1		10
Medicago polymorpha L. var. polymorpha	Fabaceae	Fodder	Casual	1957		1	1		2
Melia azedarach L.	Meliaceae	Ornamental, Timber	Invasive	1903	4	5	4		14
Melilotus albus Medik.	Fabaceae	Fodder	Naturalised	1931	1	1	2		4
Melilotus indicus (L.) All.	Fabaceae	Fodder	Casual	1926	4	2			6
Microlepia strigosa (Thunb.) K. Presl	Dennstaedtiaceae	Ornamental	Casual	1951		1			1
Microsorum scandens (G.Forst.) Tindale	Polypodiaceae	Ornamental	Casual	1954		1			1
Mimosa pudica L.	Fabaceae	Unintentional	Naturalised	1918	2		1		3
Mina lobata Cerv.	Convolvulaceae	Unintentional	Casual	1919	1	1	1		3
Mirabilis jalapa L.	Nyctaginaceae	Unintentional	Naturalised	1933	1	4	3		8
Montanoa hibiscifolia (Benth.) Standl.	Asteraceae	Ornamental	Naturalised	1946		3	1		4
Moringa oleifera Lam.	Moringaceae	Food	Casual	1943		8	4	1	13
Morus australis Poir.	Moraceae	Food, ornamental	Naturalised	1926	2	2	5		9
Myriophyllum aquaticum (Vell.) Verdc.	Haloragaceae	Ornamental	Invasive	1970			10		10
Myriophyllum spicatum L.	Haloragaceae	Ornamental	Casual	1949		1			1
Nasturtium officinale W.T. Aiton	Brassicaceae	Unintentional	Naturalised	1934	1	1	1		3
Nicandra physalodes (L.) Gaertn.	Solanaceae	Ornamental	Invasive	1917	9	9	12	1	31
Nicotiana glauca Graham	Solanaceae	Ornamental	Naturalised	1944		13	7	1	21
Nicotiana rustica L.	Solanaceae	Ornamental	Naturalised	1950		1	1		2
Nicotiana tabacum L.	Solanaceae	Crop, medicinal, ornamental	Naturalised	1870	1	1			2
Nierembergia hippomanica Miers var. violacea Millán	Solanaceae	Ornamental	Casual	1973			1		1
Oenothera indecora Cambess.	Onagraceae	Ornamental	Naturalised	1953		2	7		9
Oenothera laciniata Hill	Onagraceae	Ornamental	Casual	1935	1				1
Oenothera rosea L'Hér. ex Aiton	Onagraceae	Ornamental	Invasive	1919	1	11	9		21
Oenothera stricta Ledeb. ex Link	Onagraceae	Ornamental	Naturalised	1919	1	3	3		7
Oenothera tetraptera Cav.	Onagraceae	Ornamental	Naturalised	1949		6	4		10
Opuntia ficus-indica (L.) P. Mill.	Cactaceae	Food, Ornamental	Invasive	1959		2	1		3
Oryza sativa L.	Poaceae	Food	Casual	1966			2		2
Oxalis corniculata L.	Oxalidaceae	Unintentional	Naturalised	1972			1		1
Oxalis latifolia Kunth	Oxalidaceae	Unintentional	Invasive	1937		6	3		9
Panicum trichoides Sw.	Poaceae	Fodder	Casual	1972			1		1
Parkinsonia aculeata L.	Fabaceae	Ornamental	Naturalised	1950		1	3		4
Parthenium hysterophorus L.	Asteraceae	Unintentional	Invasive	1982			2		2
Paspalum dilatatum Poir.	Poaceae	Fodder	Casual	1919	3	2	9		14
Paspalum distichum L.	Poaceae	Fodder	Casual	1960		1	4	1	6
Paspalum notatum Flüggé	Poaceae	Fodder	Naturalised	1965			1		1
Paspalum urvillei Steud.	Poaceae	Unintentional	Naturalised	1919	2	2	13		17
Passiflora edulis Sims	Passifloraceae	Food	Invasive	1944		2	1		3
Passiflora ligularis Juss.	Passifloraceae	Unintentional	Casual	1947		5			5
Passiflora subpeltata Ortega	Passifloraceae	Ornamental	Naturalised	1971			3		3
Passiflora tarminiana Coppens & V.E. Barney	Passifloraceae	Ornamental	Casual	1976			1		1
Passiflora tripartita var. mollissima (Kunth) Holm-Niels. & P.M. Jørg.	Passifloraceae	Food	Naturalised	1920	1	1	2	1	5
Pennisetum clandestinum Hochst. ex Chiov.	Poaceae	Ornamental	Naturalised	1921	1	2	4		7
Pennisetum glaucum (L.) R.Br.	Poaceae	Food	Casual	1920	3	1	5		9
Pennisetum setaceum (Forssk.) Chiov.	Poaceae	Ornamental	Naturalised	1960		1	5	1	7
Pereskia aculeata P. Mill.	Cactaceae	Ornamental	Casual	1942		5	2		7
Persicaria capitata (Buch.-Ham. ex D. Don) H. Gross	Polygonaceae	Ornamental	Invasive	1959		1	4		5
Persicaria lapathifolia (L.) Gray	Polygonaceae	Unintentional	Invasive	1919	1	14	14		29
Persicaria limbata (Meisn.) H. Hara	Polygonaceae	Unintentional	Invasive	1931	3	8	27		38
Phalaris arundinacea L.	Poaceae	Fodder	Casual	1955		1	2		3
Phalaris minor Retz.	Poaceae	Unintentional	Casual	1925	1	4	5		10
Phyllanthus fraternus G.L.Webster	Phyllanthaceae	Unintentional	Naturalised	1964			1		1
Physalis angulata L.	Solanaceae	Unintentional	Naturalised	1947		1	4		5
Physalis lagascae Roemer & J.A. Schultes	Solanaceae	Unintentional	Casual	1958		2	2		4
Physalis peruviana L.	Solanaceae	Food	Naturalised	1906	2	2	3		7
Physalis philadelphica Lam.	Solanaceae	Food	Casual	1942		3			3
Physalis philadelphica var. immaculata Waterf.	Solanaceae	Unintentional	Naturalised	1922	3	4	2		9
Phytolacca octandra L.	Phytolocaceae	Ornamental	Naturalised	1956		1			1
Pinus elliottii Engelm.	Pinaceae	Ornamental, timber	Casual	1923	2	4	2		8
Pinus patula Schiede ex Schltdl. & Cham.	Pinaceae	Ornamental, timber	Invasive	1902	1	6	4		11
Pinus roxburghii Sarg.	Pinaceae	Ornamental, timber	Invasiv	1902	1	1			2
Pistia stratiotes L.	Araceae	Ornamental	Invasive	1948		4	7		11
Plantago lanceolata L.	Plantaginaceae	Unintentional	Naturalised	1921	1	4	1		6
Plantago major L.	Plantaginaceae	Unintentional	Naturalised	1942		2	3		5
Plantago palmata Hook. F.	Plantaginaceae	Unintentional	Casual	1920	1		2		3
Plectranthus comosus Andrews	Lamiaceae	Ornamental	Naturalised	1966			2		2
Poa annua L.	Poaceae	Unintentional	Naturalised	1934	1	5	10		16
Polycarpon tetraphyllum (L.) L.	Caryophyllaceae	Unintentional	Casual	1962			1		1
Polygonum aviculare L.	Polygonaceae	Unintentional	Naturalised	1919	1	2	2		5
Polypogon monspeliensis (L.) Desf.	Poaceae	Fodder	Casual	1968			1		1
Polypogon viridis (Gouan) Breistr.	Poaceae	Fodder	Casual	1948		1			1
Pontederia cordata L. var. lanceolata (Nutt.) Griseb.	Pontederiaceae	Ornamental	Naturalised	1975			1		1
Populus x canescens (Ait.) Smith	Salicaceae	Unintentional	Invasive	1947		1	3		4
Portulaca oleracea L.	Portulacaceae	Food	Naturalised	1975			1		1
Primula malacoides Hort.	Primulaceae	Ornamental	Naturalised	1972			2		2
Prosopis spp.	Fabaceae	Unintentional	Naturalised	1974			1		1
Prunus cerasoides D. Don	Rosaceae	Ornamental	Invasive	1950		1	6		7
Prunus serotina Ehrh. var. salicifolia (Kunth.) Koene	Rosaceae	Ornamental	Casual	1944		3	2		5
Psidium cattleianum Afzel. ex Sabine	Myrtaceae	Food, ornamental	Casual	1961		1			1
Psidium guajava L.	Myrtaceae	Food, ornamental	Invasive	1951		4	6		10
Punica granatum L.	Lythraceae	Ornamental	Casual	1931	1				1
Ranunclulus sceleratus L.	Ranunculaceae	Unintentional	Casual	1965			3		3
Raphanus raphanistrum L.	Brassicaceae	Food	Casual	1919	1	1			2
Raphanus sativus L.	Brassicaceae	Food	Casual	1918	1	1	1		3
Rapistrum rugosum (L.) All.	Brassicaceae	Unintentional	Naturalised	1958		1			1
Reseda odorata L.	Resedaceae	Ornamental	Casual	1957		1	1		2
Richardia brasiliensis Gomes	Rubiaceae	Unintentional	Invasive	1919	5	11	7		23
Richardia scabra L.	Rubiaceae	Unintentional	Invasive	1944		4	9		13
Ricinus communis L.	Euphorbiaceae	Ornamental	Invasive	1944		3	5	1	9
Rivina humilis L.	Phytolaccaceae	Ornamental	Naturalised	1962			1		1
Rubus niveus Thunb.	Rosaceae	Unintentional	Invasive	1961			6		6
Rumex acetosella L.	Polygonaceae	Unintentional	Naturalised	1935		9	3		12
Rumex crispus L.	Polygonaceae	Unintentional	Naturalised	1931	1	4	4		9
Rumex pulcher L. subsp. pulcher	Polygonaceae	Unintentional	Naturalised	1973			1		11
Sacciolepis interrupta (Willd.) Stapf	Poaceae	Unintentional	Casual	1969			6		6
Sagina apetala Ard.	Caryophyllaceae	Unintentional	Casual	1959		1	3		4
Salix babylonica L.	Salicaceae	Ornamental	Naturalised	1965			2		2
Salvia coccinea Buc'hoz ex Etl.	Lamiaceae	Ornamental	Naturalised	1917	2	4	6		12
Salvia nilotica Juss. ex Jacq.	Lamiaceae	Unintentional	Casual	1917	5	9	7		21
Salvia reflexa Hornem.	Lamiaceae	Unintentional	Casual	1955		1	1		2
Salvinia molesta Mitchell	Salviniaceae	Ornamental	Invasive	1952		10	4	3	17
Sambucus nigra subsp. canadensis (L.) R. Bolli	Adoxaceae	Ornamental	Casual	1965			1		1
Santolina chamaecyparissus L.	Asteraceae	Ornamental	Casual	1955		1	2		3
Schizolobium parahyba (Vell.) S.F.Blake	Fabaceae	Ornamental	Casual	1965			3		3
Schkuhria pinnata (Lam.) Kuntze ex Thell.	Asteraceae	Unintentional	Invasive	1918	3	15	13	3	34
Senecio vulgaris L.	Asteraceae	Unintentional	Naturalised	1970			1		1
Senna bicapsularis (L.) Roxb.	Fabaceae	Ornamental	Naturalised	1959		1	5	1	7
Senna didymobotrya (Fresen.) H.S.Irwin & Barneby	Fabaceae	Ornamental	Invasive	1921	3	8	6		17
Senna hirsuta (L.) H.S.Irwin & Barneby	Fabaceae	Ornamental	Casual	1962			1		1
Senna obtusifolia (L.) H.S.Irwin & Barneby	Fabaceae	Ornamental	Naturalised	1932	2	14	15	2	33
Senna occidentalis (L.) Link	Fabaceae	Ornamental	Invasive	1912	4	12	13		29
Senna septemtrionalis (Viv.) H.S.Irwin & Barneby	Fabaceae	Ornamental	Naturalised	1906	4	10	9		23
Senna siamea (Lam.) H.S.Irwin & Barneby	Fabaceae	Ornamental	Casual	1971			1		1
Sesbania punicea (Cav.) Benth.	Fabaceae	Fodder	Naturalised	1908	6	19	14	1	40
Setaria italica (L.) P.Beauv.	Poaceae	Food	Casual	1912	1	3	1		5
Sherardia arvensis L.	Rubiaceae	Unintentional	Casual	1964			1		1
Sida cordifolia subsp. maculata (Cav.) Marais	Malvaceae	Fibre	Invasive	1904	5	23	9		37
Silene armeria L.	Caryophyllaceae	Ornamental	Casual	1957		1			1
Silene gallica L.	Caryophyllaceae	Unintentional	Casual	1910	4	2	1		7
Sinapis arvensis L.	Brassicaceae	Unintentional	Naturalised	1915	1	1	1		3
Sisymbrium orientale L.	Brassicaceae	Unintentional	Naturalised	1956		1	1		2
Solanum americanum Mill.	Solanaceae	Unintentional	Casual	1955		1			1
Solanum betaceum Cav.	Solanaceae	Ornamental	Naturalised	1976			1		1
Solanum lycopersicum L.	Solanaceae	Food	Casual	1958		1	1		2
Solanum mauritianum Scop.	Solanaceae	Unintentional	Invasive	1976			1		1
Solanum seaforthianum Andrews	Solanaceae	Ornamental	Invasive	1918	1	5	10		16
Sonchus asper (L.) Hill	Asteraceae	Unintentional	Invasive	1917	8	10	19		37
Sorghum bicolor (L.) Moench ssp. drummondii (Steud.) deWet	Poaceae	Food	Casual	1920	3	7	3		13
Sorghum halepense (L.) Pers.	Poaceae	Food	Casual	1920	1	4	10		15
Spathodea campanulata P. Beauv.	Bignoniaceae	Ornamental	Casual	1977			1	1	2
Spergula arvensis L.	Caryophyllaceae	Unintentional	Casual	1944		1	2		3
Sphagneticola trilobata (L.) Pruski	Asteraceae	Unintentional	Naturalised	1976			2		2
Stachytarpheta cayennensis (Rich.) Vahl	Verbenaceae	Ornamental	Casual	1977			1		1
Stellaria media (L.) Vill.	Caryophyllaceae	Unintentional	Naturalised	1919	1	5	5		11
Symphyotrichum subulatum (Michx.) G.L.Nesom var. squamatum (Spreng.) S.D.Sundb.	Asteraceae	NA	Casual	1958		4	1		5
Syzygium cumini (L.) Skeels	Myrtaceae	Food	Casual	1949		3	4		7
Syzygium jambos (L.) Alston	Myrtaceae	Food	Naturalised	1953		1	3		4
Tagetes erecta L.	Asteraceae	Ornamental	Naturalised	1969			1		1
Tagetes minuta L.	Asteraceae	Unintentional	Invasive	1917	2	9	16	1	28
Taraxacum croceum Dahlst.	Asteraceae	Unintentional	Naturalised	1943		1	7		8
Tecoma stans (L.) Juss. ex Kunth	Bignoniaceae	Ornamental	Casual	1981			1		1
Tephrosia vogelii Hook.f.	Fabaceae	Unintentional	Naturalised	1919	2	7	4		13
Tipuana tipu (Benth.) Kuntze	Fabaceae	Ornamental	Casual	1967			1		1
Tithonia diversifolia (Hemsl.) A. Gray	Asteraceae	Ornamental	Naturalised	1944		4	2		6
Tithonia rotundifolia (Mill.) S.F. Blake	Asteraceae	Ornamental	Invasive	1944		3	7		10
Toona ciliata M. Roem.	Meliaceae	Ornamental, timber	Invasive	1903	4	3	3		10
Tridax procumbens L.	Asteraceae	Unintentional	Naturalised	1948		5	12		17
Trifolium campestre Schreb.	Fabaceae	Unintentional	Casual	1959		1	1		2
Trifolium dubium Sibth.	Fabaceae	Unintentional	Naturalised	1931	2	1	1		4
Trifolium repens L.	Fabaceae	Unintentional	Naturalised	1931	4	1	5		10
Verbena bonariensis L.	Verbenaceae	Ornamental	Naturalised	1924	1	1	2	1	5
Verbena brasiliensis Vell.	Verbenaceae	Unintentional	Casual	1931	2	14	14		30
Verbena litoralis Kunth	Verbenaceae	Unintentional	Casual	1958		1	1		2
Verbena rigida Spreng.	Verbenaceae	Unintentional	Casual	1974			3		3
Verbesina encelioides (Cav.) A. Gray	Asteraceae	Unintentional	Naturalised	1959		2		1	3
Vernonanthura polyanthes (Spreng.) A.J. Vega & Dematt.	Asteraceae	Unintentional	Invasive	2016				6	6
Veronica persica Poir.	Plantaginaceae	Unintentional	Casual	1959		1			1
Vicia sativa L. var. angustifolia L.	Fabaceae	Fodder	Naturalised	1926	2	6	3		11
Vicia villosa subsp. villosa	Fabaceae	Unintentional	Casual	1955		6	1		7
Wigandia urens (Ruiz & Pavon) Kunth var. caracasana (Kunth) D. N. Gibson	Hydrocharitaceae	Ornamental	Naturalised	1928	1	4	2		7
Wigandia urens var. caracasana (Kunth) D. Gibson	Boraginaceae	Ornamental	Casual	1965			1		1
Xanthium spinosum L.	Asteraceae	Unintentional	Invasive	1906	3	4	3		10
Xanthium strumarium L.	Asteraceae	Unintentional	Invasive	1942		7	6		13
Zephyranthes minuta (Kunth) D.Dietr.	Amaryllidaceae	Ornamental	Casual	1933	1	4			5
Zinnia peruviana (L.) L.	Asteraceae	Unintentional	Naturalised	1943		2	7		9
Ziziphus mauritiana Lam.	Rhamnaceae	Food	Naturalised	1950		2	2		4
