# Supplementary material for: Data on introduced plants in Zimbabwe: Floristic changes and patterns of collection based on historical herbarium records
Source: Data Brief. 2017 Sep 22;15:348–69. doi: 10.1016/j.dib.2017.09.046 (PMC5712053; doi:10.1016/j.dib.2017.09.046)
Supplement: Supplementary file 1 — Supplementary material [file mmc1.docx]

**Conflict of Interest**

There is no conflict of interest in publishing this research paper.
